# Aberrant expression of PYGB as a potential therapeutic target and its associations with immune cell infiltration in lung cancer

**DOI:** 10.3389/fimmu.2025.1536248

**Published:** 2025-05-19

**Authors:** Kai Sun, De-chang Xu, Xia Qin, Fang-fang Xie

**Affiliations:** ^1^ Department of Oncology, Ganzhou Cancer Hospital, Ganzhou, Jiangxi, China; ^2^ Department of Oncology, Liuzhou People’s Hospital, Liuzhou, Guangxi Zhuang Autonomous Region, China; ^3^ Department of Rheumatology, Ganzhou People’s Hospital, Ganzhou, Jiangxi, China

**Keywords:** PYGB, LC, prognosis, immune infiltration, biomarkers

## Abstract

**Background:**

Brain glycogen phosphorylase (PYGB) facilitates the breakdown of glycogen, thereby supplying energy to tumor cells. While PYGB expression has been documented in various tumor types, its specific function in lung cancer (LC) remains to be elucidated. This study aims to explore the potential involvement of PYGB in the initiation and progression of LC.

**Methods and results:**

We systematically analyzed PYGB in LC using data from the Cancer Genome Atlas (TCGA) and the Gene Expression Omnibus (GEO) Cancer database, employing R and various online analytical tools. Elevated PYGB expression was observed in LC and was associated with poor clinical outcomes. *In vitro* experiments, immunohistochemistry (IHC) confirmed the aberrantly high expression of PYGB in LC. The application of PYGB-siRNA significantly inhibited the proliferation, migration, and invasion of LC cells. Further analysis demonstrated correlations between PYGB expression and immune infiltration, immune checkpoint expression, tumor mutation burden, and microsatellite instability in LC.

**Conclusions:**

This study unveils that elevated PYGB expression in LC is significantly correlated with poor prognosis, potentially attributable to PYGB’s facilitation of LC cell proliferation, migration, and metastasis, as well as its significant association with the immune microenvironment.

## Introduction

In 2020, the International Agency for Research on Cancer (IACR) updated the GLOBOCAN database, revealing that lung cancer (LC) ranks as the second most prevalent cancer globally and holds the highest mortality rate among all cancers (1). Non-small cell lung cancer (NSCLC) represents the predominant clinical subtype of lung cancer. Importantly, lung adenocarcinoma (LUAD) and lung squamous cell carcinoma (LUSC) are the most frequently occurring histopathological subtypes within NSCLC (2). Owing to pioneering advancements in diagnostic technology, imaging techniques, surgical procedures, and various other domains, the management of LC has transcended the decades-long stagnation period characterized by radiotherapy and chemotherapy, ushering in a novel era of targeted therapy and immunotherapy (3). Particularly in the diagnosis and treatment of diverse solid tumors, the identification of gene targets and immune checkpoints has become an indispensable component (4). Extensive clinical studies have substantiated that early diagnosis, prompt detection of gene locus mutations, and high expression of immune checkpoints are pivotal for assessing the malignancy level, survival prognosis, and guiding clinical treatment strategies in LC (5). Nevertheless, the availability of biomarkers for clinical detection remains limited, with a notable absence of significant specificity and accuracy. Even widely utilized biomarkers, such as Programmed Death-Ligand 1 (PD-L1), tumor mutational burden (TMB), and microsatellite instability (MSI), are subject to considerable controversy (4, 6). For instance, certain studies have indicated that PD-L1 expression levels are dynamic, and there is a dearth of authentic clinical data to corroborate its efficacy in predicting immunotherapy response (7, 8). Furthermore, while high TMB tumors exhibit favorable treatment responses, the potential efficacy of related treatments in patients with low TMB cannot be unequivocally dismissed. Additionally, variations in lesion types, detection platforms, methodologies, and threshold values can influence the detection outcomes of relevant biomarkers (8). Therefore, whether considering established biomarkers with proven efficacy or novel biomarkers undergoing clinical trial validation, further corroboration through robust theoretical evidence and clinical studies is imperative to ascertain their effectiveness.

Brain Glycogen Phosphorylase (PYGB), is a rate-limiting enzyme in the process of glycogen catabolism (9). This enzyme facilitates the mobilization of glycogen, providing essential energy and metabolic intermediates that support rapid cell proliferation and survival in adverse conditions (10, 11). Moreover, the dysregulation of PYGB has been implicated in various pathologies, including cancer, where its activity can influence tumor growth and progression (12). Recent studies have highlighted the role of PYGB in metabolic reprogramming within cancer cells, particularly under hypoxic conditions commonly found in tumor microenvironments (13). Evidence suggests that increased glycogen metabolism via PYGB is crucial for maintaining cellular energy levels and preventing premature senescence in cancer cells (14, 15). The association of PYGB with different diseases underscores its potential as a therapeutic target, as modulating its activity could impact tumor metabolism and growth (14, 16). Understanding the structural and regulatory properties of PYGB, especially in comparison to other glycogen phosphorylase isozymes, can provide insights into its unique functions in the brain and its contributions to cancer biology (17). This knowledge may pave the way for novel strategies aimed at targeting PYGB in cancer treatment, potentially improving therapeutic outcomes for patients with malignancies.

PYGB is integral to the progression of various cancers, including hepatocellular carcinoma (HCC) and ovarian cancer. In HCC, PYGB is notably overexpressed and correlates with aggressive tumor phenotypes and poor prognosis. The knockdown of PYGB has been demonstrated to inhibit cell proliferation, migration, and invasion, indicating its potential as a novel prognostic biomarker and therapeutic target for HCC (18). Similarly, in ovarian cancer, aberrant autophagy regulated by the deubiquitinase PSMD14, which interacts with glycogen metabolism, facilitates cancer progression, underscoring the significance of metabolic pathways in oncogenesis (19). Moreover, the suppression of glycogen catabolism, involving the enzyme PYGB, has been demonstrated to trigger intrinsic apoptosis and enhance the efficacy of multikinase inhibitors in HCC cells. This underscores the potential of targeting glycogen metabolism as a viable therapeutic strategy in cancer treatment (20). Furthermore, the modulation of glycogen phosphorylase B by miR-101-3p in HCC indicates a complex regulatory network involving PYGB that could be leveraged for therapeutic interventions (18).

In LC, particularly non-small cell lung cancer (NSCLC), the role of PYGB is less well-defined. Nonetheless, studies have reported upregulation of PYGB in NSCLC tissues and cell lines, suggesting an association with poor patient prognosis (21). Functional assays reveal that PYGB knockdown reduces cell viability, proliferation, migration, and invasion while enhancing apoptosis. Mechanistically, the overexpression of PYGB activates the phosphoinositide 3-kinase (PI3K)/protein kinase B (Akt) signaling pathway, an effect that is effectively reversed by PYGB knockdown (21). This suggests that PYGB may play a similar role in lung cancer as it does in other cancers, acting as a potential molecular therapeutic target. Recent research indicates that metabolic reprogramming driven by PYGB facilitates tumor growth in hypoxic conditions (22), a characteristic feature of the lung tumor microenvironment. Nonetheless, a thorough analysis connecting PYGB expression with lung cancer prognosis, immune cell infiltration, or its therapeutic implications remains absent. This study aims to fill this gap by examining the role of PYGB in lung cancer progression and its interactions with the tumor immune microenvironment, thereby proposing PYGB as a novel biomarker and potential therapeutic target.

This study systematically analyzed PYGB expression and its relationship with LC prognosis using multidimensional data from the TCGA and GEO databases. To validate our findings, we performed immunohistochemical (IHC) experiments. Moreover, we assessed the effects of downregulating PYGB on the proliferation, invasion, and metastasis of LC cells. Finally, we investigated the influence of PYGB on the tumor immune infiltration microenvironment in LC. Our research unveils fresh perspectives on the pivotal role of PYGB in the intricate landscape of LC.

## Materials and methods

### Data collection and expression analysis

The pan-cancer tissue data information was obtained from the TCGA database (https://portal.gdc.cancer.gov/) and TARGET database (https://ocg.cancer.gov/programs/target/data-matrix), and the normal tissue data information was obtained from the Genotype-Tissue Expression databases (GTEx, http://gtexportal.org/) (23). Moreover, the gene expression profiles of GSE32863 (57 lung adenocarcinoma, 58 normal tissues), GSE44077 (63 lung cancer tissues, 161 normal tissues) and GSE72094 (442 lung adenocarcinoma tissues) were downloaded from GEO database (https://www.ncbi.nlm.nih.gov/geo/) to compare the expression differences of PYGB in pan-cancer and normal tissues (24). After extracting data in TPM format, we normalized it using the logarithmic transformation (log2(TPM+1)) transformation. Missing data within the databases were imputed using the missForest package in R (25). Outliers were identified through the interquartile range (IQR) method, where data points falling below Q1 - 1.5 * IQR or above Q3 + 1.5 * IQR were classified as outliers (26). These outliers were subsequently winsorized to the nearest non-outlier value. Samples were ultimately selected for further analysis after retaining samples that included both RNAseq data and clinical information.

### Prognosis analysis and establishment of nomogram

Based on the information extracted from the database, Kaplan-Meier was used to draw the survival curves of overall survival (OS), progression-free survival (PFS) and disease-specific survival (DSS) under different PYGB expression levels by R packages “DESeq2” and “limma” (27, 28). The R package “timeROC” was used to analyze the 1-year, 3-year and 5-year survival rates and draw receiver operating characteristic (ROC) curves, and the area under the curves (AUC) value under the curve was calculated (29). In addition, the survival curves of PYGB in LUAD and LUSC with different expression levels were drawn through UALCAN (https://ualcan.path.uab.edu/), GEO database and Ensemble genomic database (http://uswest.ensembl.org/index.html) (24, 30).

Initially, perform both univariate and multivariate analyses employing the Cox proportional hazards regression model. The “forestplot” package should be utilized to create a forest plot, which visually represents the P-value, hazard ratio (HR), and 95% confidence interval (CI) for each variable. Univariate and multivariate Cox regression analyses were employed to develop a nomogram based on PYGB expression levels and clinical variables such as age, gender, and pathological stage. The prognostic value of the nomogram in predicting survival risk was assessed using a calibration curve, the concordance index (C-index), and a Sankey diagram. Subsequently, informed by the results of the multivariate Cox proportional hazards model, construct a nomogram using the “rms” package to predict the overall recurrence rate at 1, 3, and 5 years.

### Tissue samples and immunohistochemistry

We collected 15 pairs of LC (12 LUSC tissues and 3 LUAD tissues) and adjacent lung tissues from Liuzhou People’s Hospital, which was reviewed and approved by the Ethics Committee (Reference No. KY2021-025-02). Patients (n=15, Age:≤55 = 3, >55 = 12; Gender: 4=Male, 11=Female; Tumor size: ≤5cm=6, >5cm=9; Smoking no=9, yes=6; Histological type: 12=LUSC, 3=LUAD; Differentiation: well=1, moderate=10, poor=1; Lymph node metastasis: no=10, yes=5) were diagnosed based on histopathological confirmation. Relevant clinical data can be found in **Supplementary Table S1**. Inclusion criteria: histologically confirmed LC, complete clinical data; exclusion criteria: unclear pathological diagnosis, incomplete clinical characteristics, having undergone over three lines of drug therapy. In the first step, the tissue was fixed in 10% formalin, embedded in paraffin, sectioned over four millimeters, dewaxed, hydrated, and repaired with antigens (1:200; ABclonal, China). A secondary antibody conjugated to horseradish peroxidase (Maxim, China) was applied to the sections followed by a 3,3′33lowedseh,ics,lt (DAB) and hematoxylin staining. By using Image-ProPlus6.0 software (Media Cybernetics, USA), we calculated the integrated optical density (IOD) for each section based on the pictures taken per slice.

### Cell culture

Human lung adenocarcinoma cells (NCI-H1975) were purchased from Institute of Basic Medical Sciences Chinese Academy of Medical Sciences, and lung squamous carcinoma cell line (NCI-H226) was provided by Stem Cell Bank, Chinese Academy of Sciences. All these cells were cultured in RPMI 1640 medium (Gibco, USA) supplemented with 10% fetal bovine serum (FBS, Gibco, USA), with additional 1% penicillin/streptomycin (Solarbio, China) at 37in humidified incubator containing 5% CO^2^.

### siRNA transfection

The small interfering RNA (siRNA) sense used were as follows: siPYGB#1(sense:5’-CCAAGCGCAUUUAUUAUCUTT-3’, antisense:5’-AGAUAAUAAAUGCGCUUGGTT-3’); siPYGB#2(sense:5’-GGGUCCUGUAUCCAAAUGATT-3’, antisense:5’-UCAUUUGGAUACAGGACCCTT-3’); siPYGB#3(sense:5’-GUGAGAACCUGUUUCGAGATT-3’, antisense:5’-UCUCGAAACAGGUUCUCACTT-3’). The sense of negative control RNA (NC) was as follows: siNC (sense:5’-UUCUCCGAACGUGUCACGUTT-3’, antisense: 5’-ACGUGCACGUUCGGAGAATT-3’). NCI-H1975 and NCI-H226 cells were seeded at 3x105 cells/well in 6-well plates 1 day before transfection until 80-90% confluence. Cells were transfected with siRNA or NC, using Lipo transfection reagent (KeyGEN, China), according to the manufacturer’s instructions. The final siRNA concentration was 50 nM. Total RNA or protein was extracted 48h after transfection for analysis.

### Real−time fluorescence quantitative PCR

Total RNAs were isolated using GeneJET RNA Purification Kit (Thermo Fisher Scientific, Inc.), according to the manufacturer’s protocol. RNA was transcribed into cDNA using RevertAid Master Mix Transcriptase kit (Thermo Fisher Scientific, Inc.). 40 ng of each cDNA sample was then analyzed via qRT-PCR using PowerUpd SYBRr Green Master Mix (Thermo Fisher Scientific, Inc.) with LightCycler 480 II instrument (Roche, USA). The relative gene expression was analyzed by 2 -△△CT Method. The primer sequences used were as follows: PYGB (forward: 5’-ACGCAGCAGCACTACTAC-3’; reverse, 5’-TCGCAGGCATTCTGAAGG’); β’-TCGC (forward: 5’-TGCGTGACATTAAGGAGAAGC-3’; reverse: 5’-GGAAGGAAGGCTGGAAGAGT-3’).

### Western blot analysis

After the transfection, cells were washed twice with PBS and lysed on ice in RIPA lysis buffer. The protein amounts were determined using BCA protein assay kit (Epizyme Biotech, Shanghai, China). Samples were loaded and separated by 10% SDS-PAGE and transferred to a nitrocellulose membrane (Millipore, USA). Membranes were blocked with 5% skim milk for 1h and incubated overnight at 4te with primary antibodies (PYGB, 1:1000; GAPDH, 1:3000; Proteintech, China). The membranes were washed in TBST 3 times for 10 min each. Then they were incubated with horseradish peroxidase conjugated goat anti-rabbit IgG (H+L) antibodies (1:2000; Proteintech, China). The membrane was washed again in the same way, and developed.

### Cell proliferation assay

Cell proliferation was measured using the CCK-8 kit (Dojindo, Japan). After transfection, cells were seeded into 96-well plates (3000 cells/well). Subsequently, 10μL CCK-8 solution was added to each well at different time points (24h, 48h, 72h, and 96h), followed by incubation at 37°C for 1h. The optical density (OD) value of each well was measured at 450 nm with a microplate reader (VarioskanLUX, USA).

### Wound healing assay

Transfected cells were seeded into 12-well plates and allowed to grow to 90% confluence. A 200uL sterile pipette tip was used to scratch the cell layer. The cell surface was gently washed to remove cell debris using serum-free medium. Continue by incubating the cells with the serum-free medium. The cells were photographed under a microscope and the location of the scratches was recorded. Finally, the cell migration rate was calculated by comparing the wound width at 0h and 24h.

### Transwell migration and invasion assay

A 24-well Transwell chamber with 8 µm pore size membranes (Corning, USA) was prepared with or without 80uL Matrigel (Corning, USA). Non-serum medium with transfected cells (NCI-H1975, 6CI-H1975,d NCI-H226, 8CI-H226,d were added to the upper inserts, while the bottom chambers were filled with 600uL medium with 20% FBS. After 24h culture, 4% paraformaldehyde was used to fix the lower chambers for 30min, followed by staining with 0.1% crystal violet for 15min. The images from five high power fields per membrane were captured to calculate the numbers of migration cells or invasion cells.

### Immune correlation analysis

The R package “immunedeconv,” “estimate,” “ggplot2,” “pheatmap,” and “ggstatsplot” were used to analyze the correlation between PYGB and immune cell infiltration(six of the latest algorithms, including xCell, CIBERSORT, EPIC, TIMER, MCP-counter, and quanTIseq), stromal, immune, and estimate scores, sixty common immune checkpoint molecules(Inhibitory [24], Stimulatory [36]), 150 marker genes are identified for five immune pathways (chemokine [41], receptor [18], MHC [21], immunoinhibitor [24], and immunostimulator [46]), Tumor Mutational Burden (TMB) and Microsatellite Instability (MSI) (31–33). The statistical analysis information was visualized by R version 4.0.3.

### Statistical analysis methods

Fold-change (FC), Hazard ratio (HR), and P-values of Log-rank test were used to analyze data. Statistical analysis was performed using R software, and Spearman correlation analysis, Pearson correlation analysis, and Wilcoxon test were used to verify the correlation between specific variables. Survival analysis was performed using Kaplan-Meier curve and log-rank test, with *p* value or log-rank *p* value <0.05 as statistically significant. *P* was represented by * in the significance difference diagram, * represents *P*<0.05, ** represents *P*<0.01, *** represents *P*<0.001, and **** represents *P*<0.0001.

## Results

### Assessment of PYGB expression in LC and normal tissues

**Figure 1:**
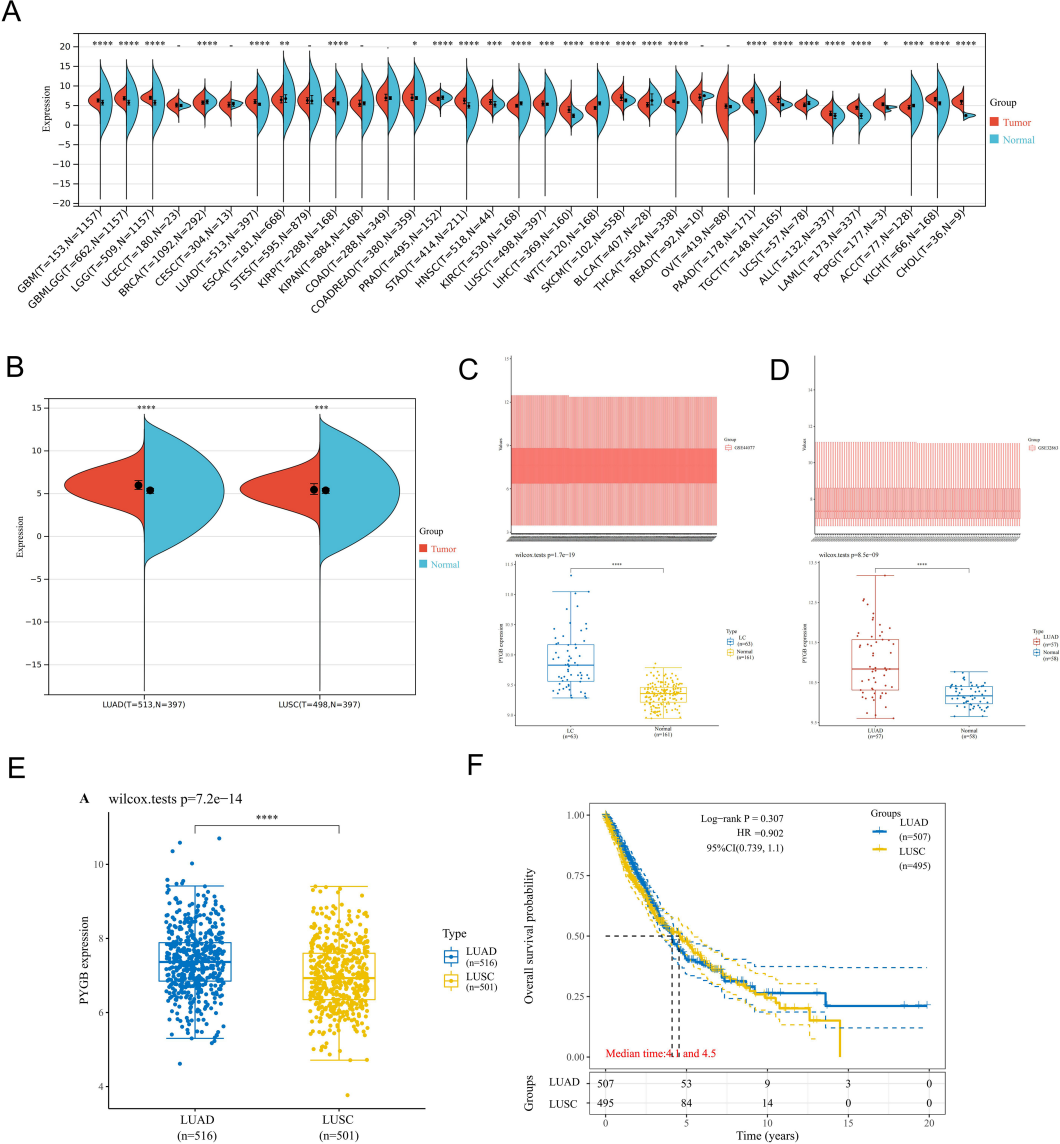
Expression analysis of PYGB. **(A)** Differential expression analysis of PYGB between pan-cancer tissues and adjacent normal tissues in TCGA and GETx database. **(B)** Differential expression analysis of PYGB between tumor tissues and normal tissues in LUAD and LUSC based on TCGA and GETx database. **(C)** Differential expression analysis of PYGB between LC tumor tissues and normal tissues based on GSE44077. **(D)** Differential expression analysis of PYGB between LUSC tumor tissues and normal tissues based on GSE32863. **(E)** Differential expression analysis of PYGB between LUAD and LUSC. **(F)** Differential prognostic analysis of PYGB between LUAD and LUSC. * *p*<0.05, ** *p*<0.01, *** *p*<0.001, **** *p*<0.0001. PYGB, Brain glycogen phosphorylase; LC, lung cancer; TCGA, The Cancer Genome Atlas; GEO, Gene Expression Omnibus; LUAD, Lung adenocarcinoma; LUSC, Lung squamous cell carcinoma.

To determine the mRNA expression profile of PYGB in pan-cancer, 34 pan-cancer tissues were extracted from TCGA and TARGET databases, and they were matched with normal tissues in GTEx database. The differences in the expression of PYGB in pan-cancer tissues and normal tissues were analyzed. It was found that the expression of PYGB in many cancer tissues was higher than that in normal tissues (GBM (Glioblastoma), GBMLGG (Glioblastoma and Lower Grade Glioma), LGG (Lower Grade Glioma), BRCA (Breast Cancer), LUAD (Lung Adenocarcinoma), KIRP (Kidney Papillary Cell Carcinoma), STAD (Stomach Adenocarcinoma), HNSC (Head and Neck Squamous Cell Carcinoma), KIRC (Kidney Renal Clear Cell Carcinoma), LUSC (Lung Squamous Cell Carcinoma), LIHC (Liver Hepatocellular Carcinoma), PAAD (Pancreatic Adenocarcinoma), etc., **Figure 1A**). PYGB expression was significantly elevated in tumor tissues compared to normal controls across both subtypes: LUAD (TCGA-LUAD: 513 tumors vs. 379 normal) and LUSC (TCGA-LUSC: 486 tumors vs. 379 normal). (**Figure 1B**). Subsequently, the GSE32863 and GSE44077 data sets in GEO database were used for further analysis, and it was discovered that the expression of PYGB in LC tissue was significantly higher compared to normal tissue (**Figures 1C, D**), which was consistent with the differential expression shown in TCGA databases. On the other hand, the expression of PYGB in LUAD was significantly higher than that in LUSC (**Figure 1E**).

### Genetic expression and variation of PYGB in LC

**Figure 2:**
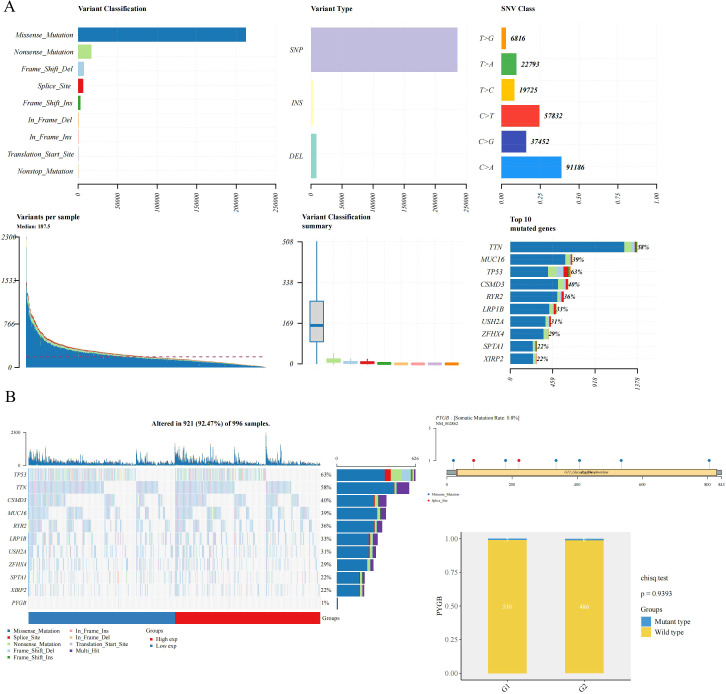
The mutation frequency and classification of PYGB in LC. **(A)** Gene mutation characteristic of LC and in TCGA database; **(B)** The somatic mutation rate of the PYGB gene was 0.8% in LC. PYGB, Brain glycogen phosphorylase; LC, lung cancer; TCGA, The Cancer Genome Atlas.

A detailed examination of PYGB genetic alterations in lung cancer is presented in **Figure 2**. As illustrated in **Figure 2B**, genomic mutations in PYGB were identified in 92.47% (921 out of 996) of LC samples. Missense mutations constituted the predominant variant classification among these genetic alterations (**Figure 2B**). At the nucleotide level, single nucleotide polymorphisms (SNPs) were the most prevalent mutation type, with C>A substitutions being the most frequent among all single nucleotide variants (SNVs) (**Figure 2A**). Notably, the overall somatic mutation frequency of PYGB across LC samples was calculated at 0.8% (**Figure 2B**).

### PYGB protein expression analysis in LC

To verify the aforementioned results, an IHC (Immunohistochemical) analysis was conducted to assess the expression of PYGB in 15 pairs of LC tumor tissues and their adjacent normal tissues. In the IHC staining analysis, PYGB proteins were predominantly localized within the cytoplasm of LC cancer cells, with brown staining serving as an indication of positive staining. (**Supplementary Figures S1C, D**). In normal tissues, PYGB proteins were either weakly expressed or not expressed (**Supplementary Figures S1A, B**). After immunohistochemical analysis, it was found that the PYGB protein levels, as determined by the value of IOD, were significantly elevated in LC tissues when compared to adjacent non-tumor tissues. (*P* < 0.001) (**Supplementary Figure S1E**).

### Prognostic value of PYGB in LC

**Figure 3:**
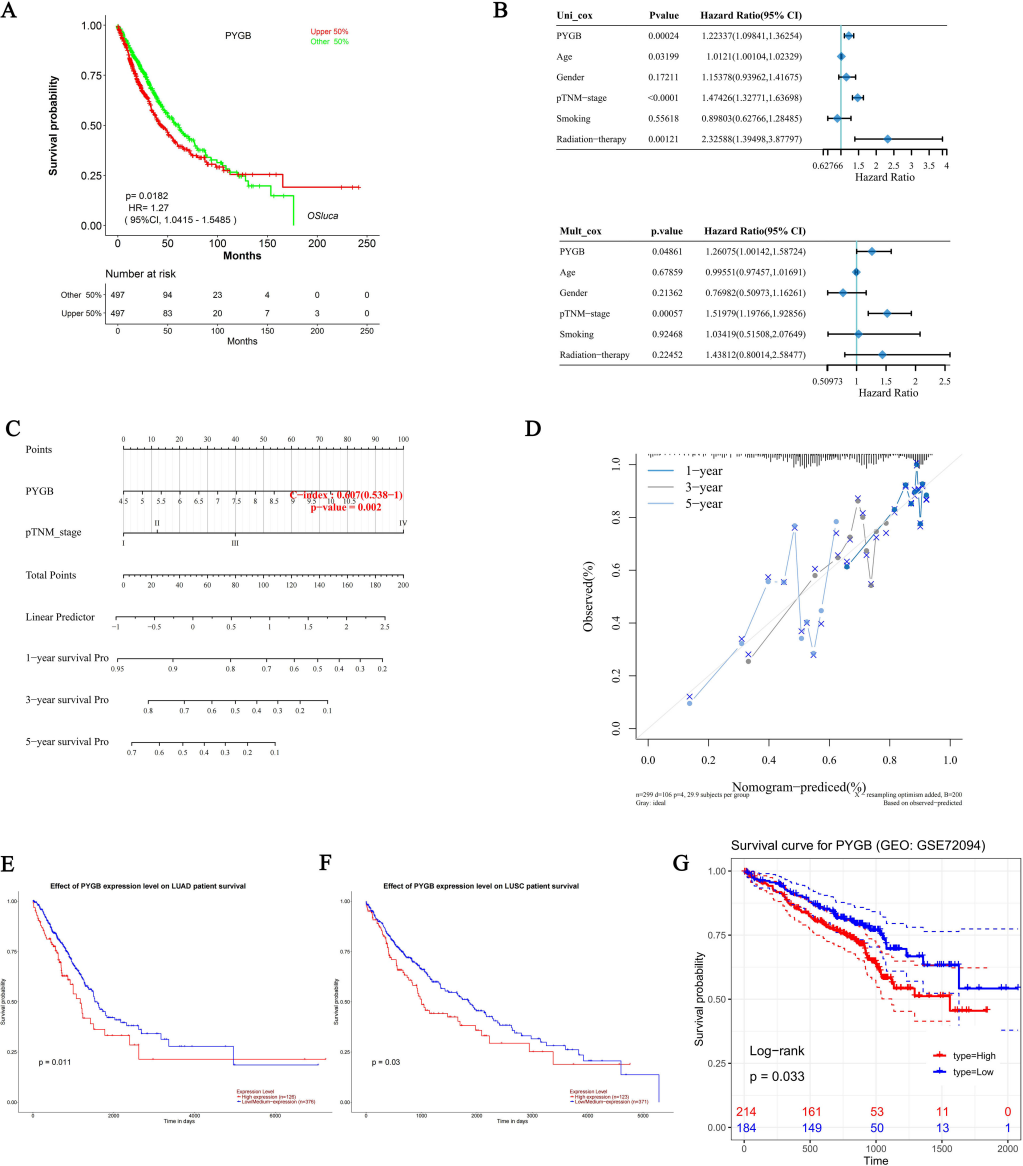
Prognostic analysis of PYGB in LC. **(A)** Prognostic analysis of PYGB mRNA expression levels in LC in the TCGA database. **(B)** Hazard ratio and p-value of constituents involved in univariate and multivariate Cox regression and some parameters of PYGB in LC. **(C)** Nomogram to predict the 1-year, 3-year, and 5-year overall survival rate of LC patients. **(D)** Calibration curve for the overall survival nomogram model in the discovery group. A dashed diagonal line represents the ideal nomogram. **(E)** Effect of PYGB expression level on LUAD patient survival. **(F)** Effect of PYGB expression level on LUSC patient survival. **(G)** Survival analysis of PYGB in LC based on the GES72094 dataset. PYGB, Brain glycogen phosphorylase; LC, lung cancer; TCGA, The Cancer Genome Atlas; GEO, Gene Expression Omnibus; LUAD, Lung adenocarcinoma; LUSC, Lung squamous cell carcinoma.

Using the KM plotter, we investigated the correlation between PYGB expression and survival in the TCGA cohort of LC patients, and the results showed that high PYGB expression was significantly associated with a worse prognosis in patients with LC (overall survival [OS], HR = 1.27, *P* = 0.0182) (**Figure 3A**). PYGB and pTNM stage were found to be independent variables that influenced the prognosis of LC with both univariate and multivariate Cox analyses (**Figure 3B**). We developed a nomogram based on PYGB and pTNM stages to predict LUSC patient survival at 1-year, 3-years, and 5-years (**Figures 3C**). The overall survival (OS) rates at 1-year, 3-year, and 5-year intervals were accurately predicted in comparison to an ideal model across the entire cohort (**Figure 3D**). Furthermore, we analyzed the correlation between PYGB expression and the prognosis of LUAD and LUSC using UALCAN, revealing that elevated PYGB mRNA expression is linked to poorer outcomes in these LUAD or LUSC patients. (*P* = 0.011; *P* = 0.03) (**Figures 3E, F**). Using the GEO dataset GSE72094, we also found that high expression of PYGB in LC patients indicates a poor prognosis (**Figure 3G**). However, there was no significant difference in the prognostic value of PYGB expression levels between LUAD and LUSC (**Figure 1F**).

### PYGB promotes the proliferation, migration, and invasion of LC cells

**Figure 4:**
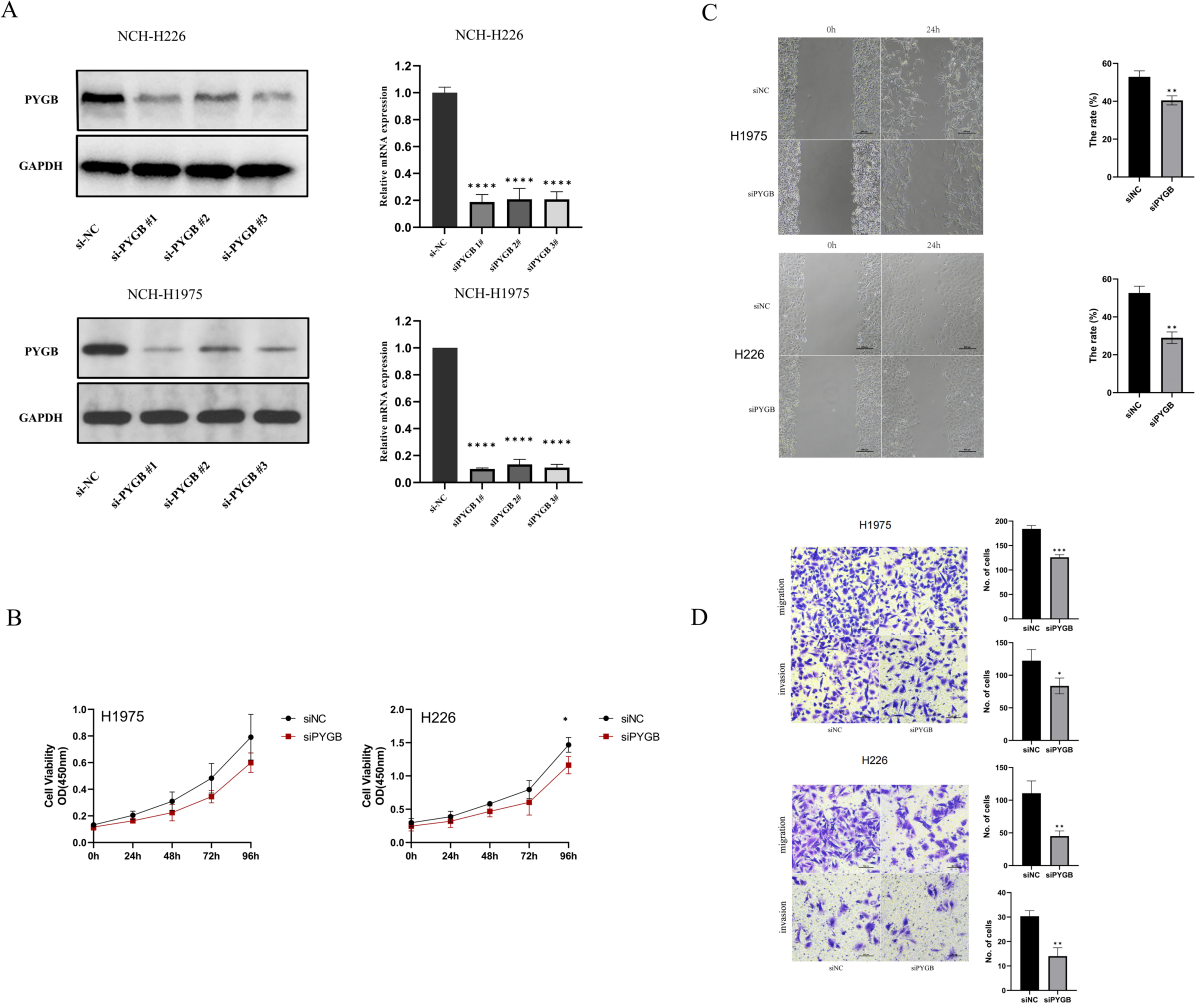
**(A)** PYGB siRNA significantly decreased the expression of PYGB. Western blot results and RT-qPCR results of the expression of PYGB in four groups (si-NC, si-PYGB-1, si-PYGB-2, si-PCCB-3) in NCI-H226 cells line and NCI-H1975 cells line. **(B)** Effect of PYGB knockdown on proliferation of LUAD and LUSC cells. CCK-8 assays detected silencing PYGB reduced the proliferative capabilities of NCI-H1975 cells line and NCI-H226 cells line. **(C)** Effect of PYGB knockdown on migration of LUAD and LUSC cells. The wound healing assay detected that the knockout of PYGB reduced the migration ability of the NCI-H1975 cell line and the NCI-H226 cell line. **(D)** Effect of PYGB on cell invasion and migration of lung cancer cells. Results of transwell assay evaluating showing Cell migration and invasion ability of NCI-H1975 cells and NCH-H226 cells decreased after PYGB-siRNA transfection. (×20 magnification). Statistical analysis was based on mean ± SD. The si-PYGB refers to siRNA transfection of PYGB. The si-NC refers to siRNA transfection of nonspecific control. *p < 0.05, **p < 0.01; ***p < 0.001; ****p < 0.0001. PYGB, Brain glycogen phosphorylase; LC, lung cancer; LUAD, Lung adenocarcinoma; LUSC, Lung squamous cell carcinoma.

To explore the role of PYGB in LC proliferation, migration, and invasion *in vitro*, we constructed siRNA targeting PYGB in Human lung squamous carcinoma cells (NCI-H226) line and Human lung adenocarcinoma cells (NCI-H1975) line (**Figure 4A**). According to western blot and RT-qPCR results, PYGB expression was markedly decreased in the three si-PYGB groups relative to the si-NC groups in NCI-H226 and NCI-H1975 cells (**Figure 4A**). The si-PYGB, which had the strongest capacity to inhibit PYGB expression, was utilized for the subsequent experiment. CCK-8 assays showed that silencing PYGB significantly reduced the proliferative capabilities of NCI-H226 cells line (*P <*0.05) (**Figure 4B**). Moreover, the silencing of PYGB was associated with a decrease in the proliferative capacity of the NCI-H1975 cell line; however, this reduction did not reach statistical significance (**Figure 4B**). This observation may indicate that, in contrast to lung adenocarcinoma cells, PYGB exerts a more pronounced influence on the proliferative capabilities of lung squamous carcinoma cells. The wound healing assay demonstrated that the knockout of PYGB reduced the migration ability of the NCI-H226 cell line and the NCI-H1975 cell line (*P <*0.01) (**Figure 4C**). As well, when PYGB was silenced, the migration and invasion abilities of the NCI-H226 and NCI-H1975 cell lines were significantly reduced in transwell migration and invasion assays. (*P <*0.05) (**Figure 4D**). These results collectively support the notion that PYGB promotes the proliferation, migration, and invasion of LC cells.

### The correlations between expression levels of PYGB and immune cell infiltration in LC

**Figure 5:**
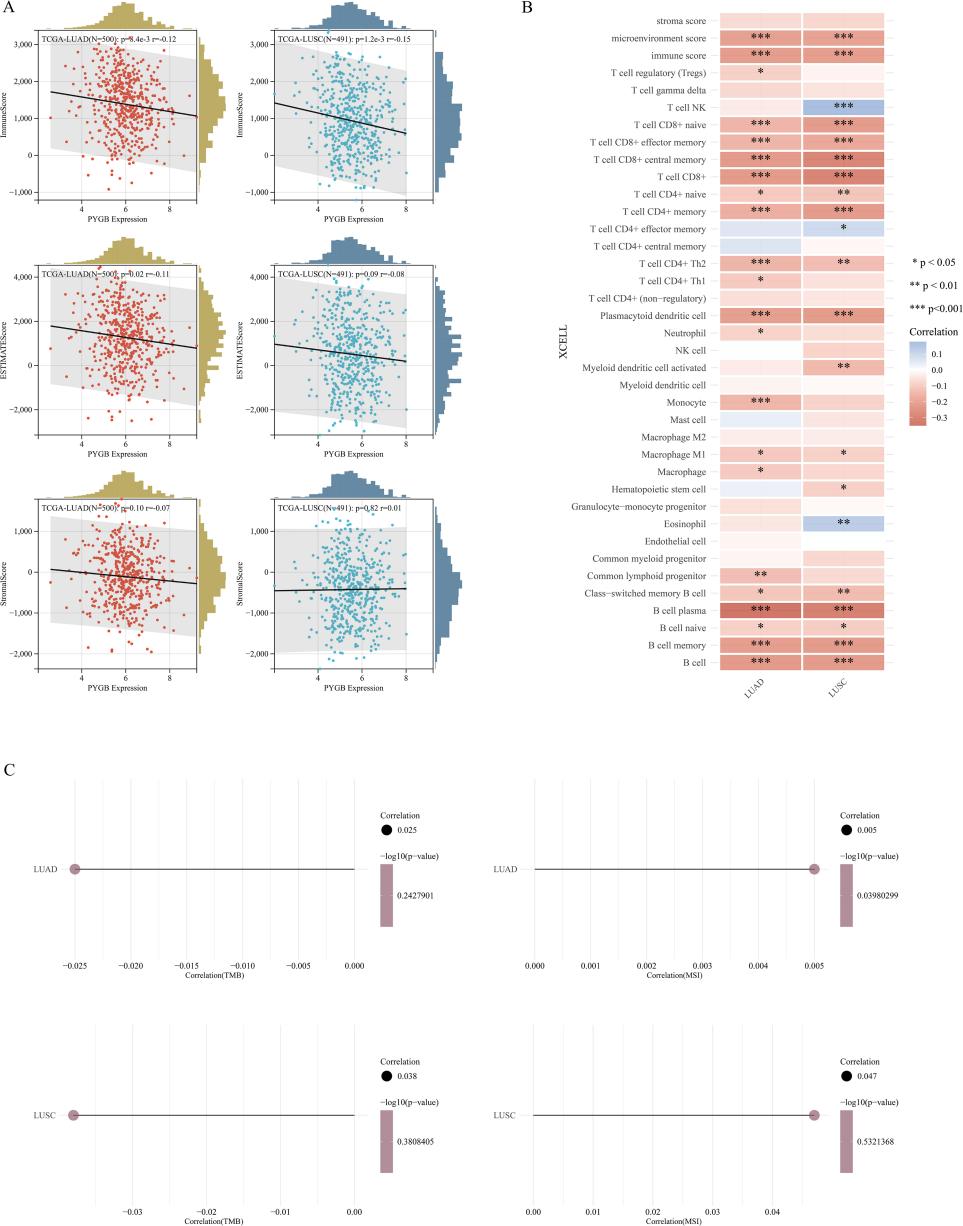
**(A)** Correlation of PYGB with tumor microenvironment scores using algorithm of ESTIMATE database: association of PYGB with immune score, stromal score, and ESTIMATE score in LUAD and LUSC. **(B)** Correlation of PYGB expression level with immune infiltration in LUAD and LUSC using algorithm of XCELL database. **(C)** Correlation between PYGB and TMB and MSI in LUAD and LUSC. *p < 0.05, **p < 0.01, ***p < 0.001. PYGB, Brain glycogen phosphorylase; LUAD, Lung adenocarcinoma; LUSC, Lung squamous cell carcinoma; TMB, Tumor mutation burden; MSI, Microsatellite instability. ESTIMATE, Estimation of STromal and Immune cells in MAlignant Tumors using Expression data.

Studies have found that tumor-infiltrating lymphocytes (TILs) are independent predictors of cancer stage, grade, and lymph node status (34, 35). We used the ‘ESTIMATE’ function in the R package to analyze the correlations between stromal scores, immune scores, and ESTIMATE scores with PYGB expression in LUAD and LUSC. Notably, in both LUAD and LUSC, PYGB expression exhibited a significant negative correlation with Immune Score (*P <*0.01) (**Figure 5A**). In LUAD, a negative correlation was observed between PYGB and ESTIMATE Score (*P <*0.05) (**Figure 5A**). In LUSC, PYGB expression negatively correlated with ESTIMATE Score, and in LUAD, it negatively correlated with Stromal score, but neither was statistically significant (*P >*0.01) (**Figure 5A**), requiring further validation.

**Figure 6:**
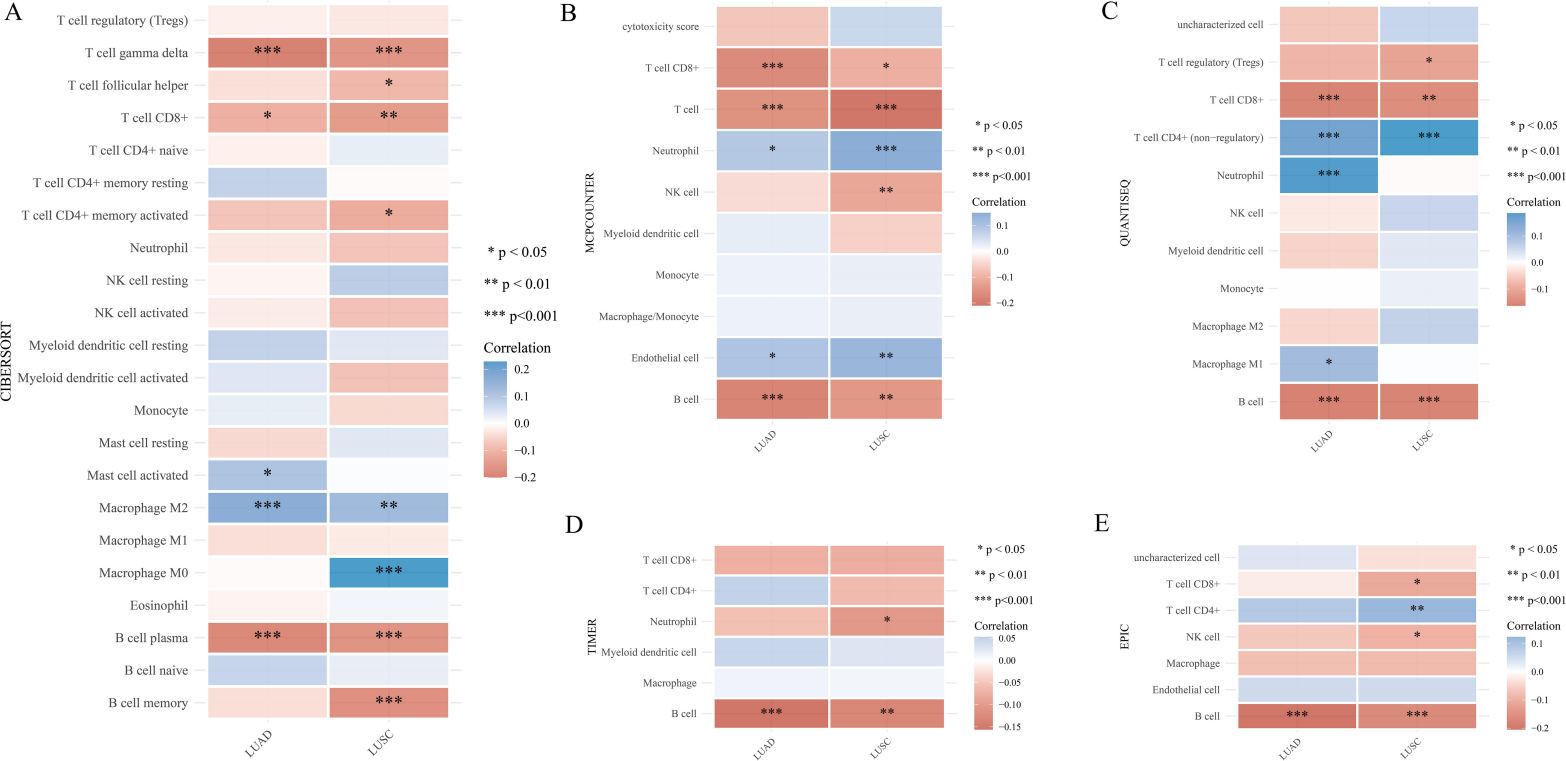
**(A)** Correlation of PYGB expression level with immune infiltration in LUAD and LUSC using algorithm of CIBERSORT database. **(B)** Correlation of PYGB expression level with immune infiltration in LUAD and LUSC using algorithm of MCP-counter database. **(C)** Correlation of PYGB expression level with immune infiltration in LUAD and LUSC using algorithm of quanTIseq database. **(D)** Correlation of PYGB expression level with immune infiltration in LUAD and LUSC using algorithm of TIMER database. **(E)** Correlation of PYGB expression level with immune infiltration in LUAD and LUSC using algorithm of EPIC database. *p < 0.05, **p < 0.01, ***p < 0.001. PYGB, Brain glycogen phosphorylase; LC, lung cancer; LUAD, Lung adenocarcinoma; LUSC, Lung squamous cell carcinoma; TCGA, The Cancer Genome Atlas; TMB, Tumor mutation burden; MSI, Microsatellite instability.

Furthermore, we utilized XCELL to investigate the potential correlation between PYGB expression and immune cell infiltration in LUAD and LUSC. Expression levels of PYGB were significantly negatively associated with T cell CD8+, T cell CD8+ naive, T cell CD8+ effector memory, T cell CD8+ central memory, T cell CD4+ memory, T cell CD4+ Th2, Plasmacytoid dendritic cell, B cell, B cell plasma, B cell memory, among others in LUAD and LUSC (*P <*0.001) (**Figure 5B**). On the contrary, PYGB expression was only positively associated with T cell NK and Eosinophil in LUSC (*P <*0.01) (**Figure 5B**). The results showed that the expression of PYGB in LUAD and LUSC showed significant correlation with the immune infiltrate (*P <*0.001) (**Figure 5B**). To further validate our findings, we employed multiple immune infiltration analysis tools, including CIBERSORT, EPIC, TIMER, MCP-Counter, and QuanTIseq (31). These algorithms collectively demonstrate robust concordance in characterizing the tumor immune microenvironment, thereby reinforcing the consistency of our observations across different computational frameworks (**Figure 6**).

### TMB, MSI, immune checkpoints, and immune regulatory genes analysis of PYGB in LUAD and LUSC

The efficacy of immune checkpoint blockade (ICB) therapy is influenced not merely by immune cell infiltration but also by TMB, MSI, immune checkpoints, and immune regulatory genes. We deeply explored the correlation between PYGB expression and TMB, MSI, and found that PYGB expression was negatively correlated with TMB in LUAD and LUSC (Correlation LUAD: -0.025, LUSC: -0.038) (**Figure 5C**), and PYGB expression was positively correlated with MSI in LUAD and LUSC (Correlation LUAD: 0.005, LUSC: 0.047, *P*=0.039) (**Figure 5C**). The result, however, was not statistically significant.

**Figure 7:**
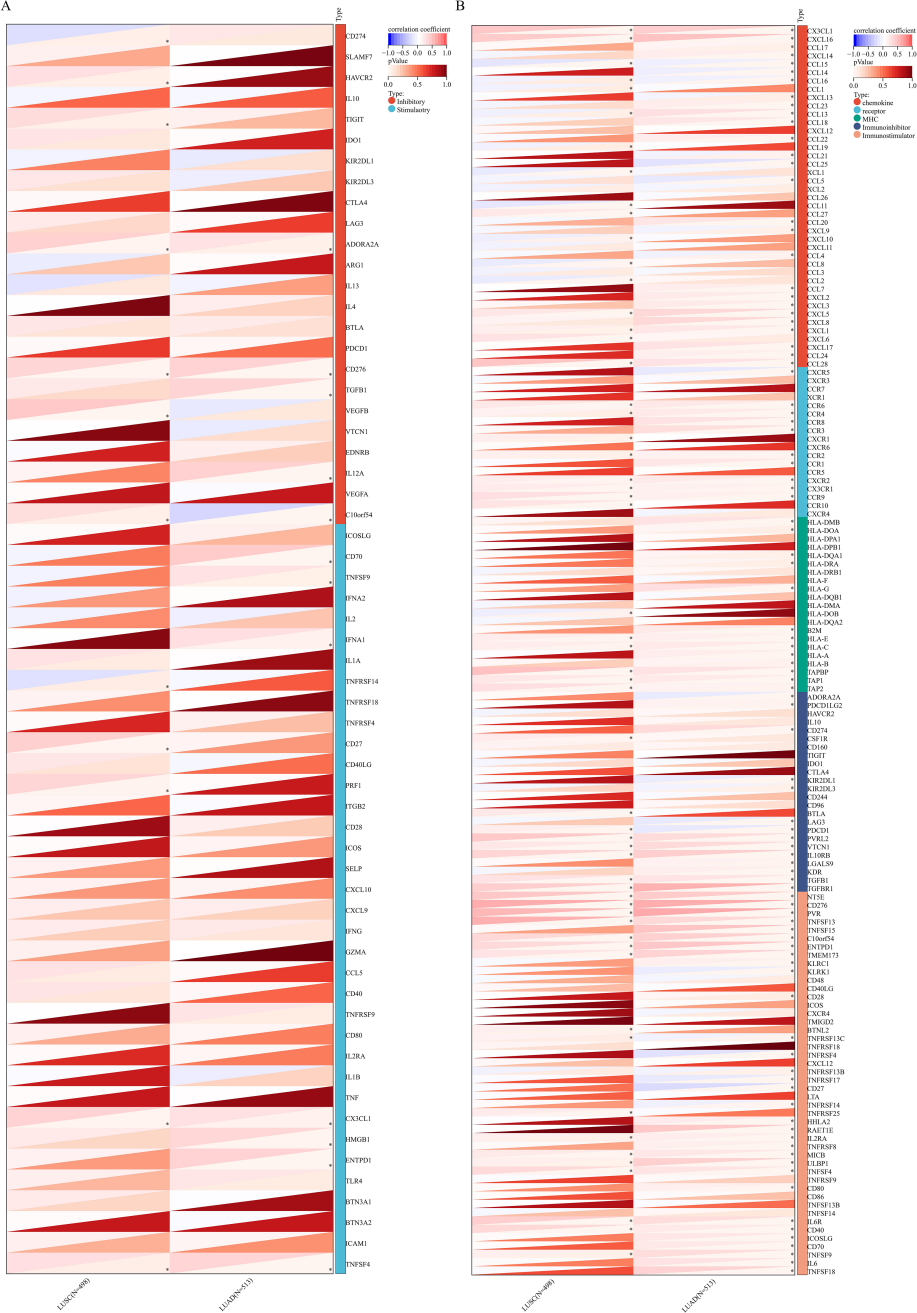
**(A)** Correlation between PYGB expression and immune checkpoint genes in LUAD and LUSC. **(B)** Correlation between PYGB expression and immune regulatory genes in LUAD and LUSC. *p < 0.05, **p < 0.01, ***p < 0.001. PYGB, Brain glycogen phosphorylase; LUAD, Lung adenocarcinoma; LUSC, Lung squamous cell carcinoma.

To further confirm the close relationship between PYGB expression and immune microenvironment, we systematically examined the associations between PYGB expression and immune checkpoint genes, immune regulatory genes in both LUAD and LUSC. Interestingly, in LUAD, PYGB expression exhibited strong positive correlations with ADORA2A, CD276 (B7-H3), TGFB1, IL12A, CD70, TNFSF9, IFNA1, CX3CL1, HMGB1, ENTPD1, and TNFSF4, while showing a negative correlation with C10orf54 (VSIR) (*P <*0.05) (**Figure 7A**). In LUSC, PYGB positively correlated with HAVCR2 (TIM-3), TIGIT, ADORA2A, CD276, VEGFB, C10orf54, CD27, PRF1, CX3CL1, and TNFSF4, but inversely correlated with CD274 (PD-L1) and TNFSF14 (LIGHT). These findings suggest that PYGB may interact with key immune regulators across LC subtypes, potentially serving as a predictive biomarker or therapeutic target to modulate immune checkpoint pathways in lung cancer immunotherapy. Subsequently, we further explored the correlation between PYGB expression and immune regulatory genes. Our findings revealed that PYGB expression exhibited significant associations with most immune regulatory genes in both LUAD and LUSC with particularly pronounced correlations observed in LUAD (*P <*0.05) (**Figure 7B**). This may suggest that PYGB plays a key role in the tumor immune infiltration microenvironment in lung cancer.

## Discussion

PYGB is a rate-limiting enzyme of glycogen catabolism, which has been confirmed by multiple studies to be involved in the occurrence and development of tumors (14). World-wide, LC is the most prevalent tumor and contributes the most to death from cancer (3). LC patients now have access to immunotherapies as a standard treatment option. LC prognosis and immunotherapy effectiveness are linked to the immune microenvironment of the tumor (8). Understanding the dynamics of the immune microenvironment in LC is essential for developing effective therapeutic strategies. This study investigated the intricate relationship between PYGB and LC, as well as the immune microenvironment.

We first used the TCGA and TARGET databases to reveal that PYGB exhibits elevated expression levels in many tumor samples when compared to normal tissues. Our finding aligned with prior research that has established comparable expression patterns of PYGB in ovarian and liver cancers (18, 36). Additionally, it was discovered that the PYGB expression of normal and tumor tissues in LUAD and LUSC differed significantly. For further analysis, GSE32863 and GSE44077 data sets were analyzed in the GEO database, and the expression of PYGB in tumor tissue was significantly higher than in normal tissue, which was consistent with the finding. According to *in vitro* experiments, immunohistochemical (IHC) analysis found that the PYGB protein level significantly increased in LC tissues when compared to adjacent non-tumor tissues. On the other hand, the significantly high expression of PYGB in LUAD as compared to LUSC may indicate a tissue-specific pattern of PYGB expression in LC.

Subsequently, we investigated to assess the influence of elevated PYGB expression on the prognosis of patients with LC. Analysis using the KM plotter revealed a significant association between elevated PYGB expression and poorer prognosis in LC patients within the TCGA cohort and GEO dataset. Both univariate and multivariate Cox analyses found that PYGB and pTNM stage were independent variables influencing LC prognosis. A nomogram incorporating PYGB and pTNM stages effectively predicted 1-year, 3-year, and 5-year OS rates for LC patients, demonstrating accuracy comparable to an ideal model across the entire cohort. Using UALCAN online tools, we analyzed the correlation between PYGB expression and the prognosis of LUAD and LUSC patients. Our analysis indicates that elevated PYGB mRNA expression is associated with poorer outcomes. In early investigations, PYGB has been identified as a pivotal enzyme in glycogen catabolism and has been implicated in the progression of various tumors (21). In NSCLC, elevated expression levels of PYGB correlate with unfavorable patient prognoses (21). Moreover, PYGB facilitates cellular proliferation and migration through the activation of the PI3K/AKT signaling pathway, underscoring its significant role in NSCLC progression (21). Additional studies suggest that the heightened expression of PYGB in lung cancer may be linked to smoking-related genetic alterations, particularly in LUSC, where PYGB may function as a biomarker associated with smoking (37). The research also indicates that silencing PYGB can impede the advancement of NSCLC, proposing that PYGB could serve as a novel biomarker and a potential molecular therapeutic target for this malignancy (37).Due to these findings, it is speculated that PYGB might promote cancer similar to early studies (38).

Since the abnormal expression of PYGB has been shown to be associated with poor prognosis in LC, it is important to understand its underlying mechanisms. Therefore, we conducted a series of *in vitro* experiments to explore the role of PYGB in LC proliferation, migration, and invasion. First, we constructed siRNA targeting PYGB in human lung squamous carcinoma (NCI-H226) cell line and human lung adenocarcinoma (NCI-H1975) cell line. Silencing PYGB significantly reduced the proliferative capabilities of the NCI-H226 cell line and NCI-H1975 cell line in CCK-8 assays. In wound healing assays, NCI-H226 and NCI-H1975 cells with knockout of PYGB showed reduced migration capacity. Likewise, NCI-H226 and NCI-H1975 cell lines with silenced PYGB showed significantly reduced migration and invasion abilities in transwell migration assays. Our experiments confirmed that PYGB promoted the proliferation, invasion, and metastasis of LC cells. In previous literatures, PYGB have been reported to be upregulated in ovarian cancer and hepatocellular carcinoma, and this dysregulation promotes ovarian cancer cell and hepatocellular carcinoma cell proliferation, invasion, migration and drug resistance (18, 36). Nonetheless, the precise mechanisms involved have yet to be elucidated.

Tumor growth, invasion, and metastasis have been shown to be influenced by the tumor immune microenvironment (34, 39, 40). Understanding the composition of immune cells in tumor tissues can shed light on novel approaches to cancer treatment and enhance the efficiency of ICB therapy. Given that LC is a highly immunogenic tumor, we analyzed the relationship between immune-related scores and PYGB expression. in LC. Based on ESTIMATE algorithms, PYGB expression in LUAD and LUSC showed a negative correlation with immune scores, and ESTIMATE scores. Known as the immune score, it reflects the number and activity of immune cells within the tumor microenvironment (TME) (41). Patients demonstrating an elevated immune score showed increased sensitivity to neoadjuvant chemoradiotherapy and enhanced survival outcomes (42). In the context of LC, elevated PYGB expression might contribute to an immune-suppressed TME by modulating the metabolic environment and influencing the recruitment and function of immune cells. Stromal scores have the potential to identify targets for therapies directed at the stromal component and to predict patient outcomes. The ESTIMATE scores integrate both stromal and immune scores to deduce the comprehensive composition of the tumor microenvironment. Low ESTIMATE scores (high tumor purity) are associated with aggressive phenotypes and resistance to immunotherapy (39). PYGB’s role in sustaining tumor cell autonomy might reduce dependence on stromal support and driving purity. Therefore, we proposed the hypothesis that the aberrant expression of PYGB might directly or indirectly affect the immune environment in LC, consequently contributing to an unfavorable prognosis.

The relationship between PYGB expression and various immune cell types in LC is an intriguing area of study, particularly given the complex interactions between tumor cells and the immune system. Further investigation using XCELL revealed that the expression levels of PYGB were significantly negatively correlated with various immune cell infiltration levels, including T cell CD8+, naive T cell CD8+, effector memory T cell CD8+, central memory T cell CD8+, memory T cell CD4+, Th2 T cell CD4+, plasmacytoid dendritic cells, B cells, plasma B cells, and memory B cells, among others, in both LUAD and LUSC. CD8+ T cells, which include naive, effector memory (TEM), and central memory (TCM) subsets, play a crucial role in anti-tumor immunity by directly killing cancer cells (43). The negative correlation of PYGB with these CD8+ T cell subsets suggested that higher PYGB expression might be associated with reduced CD8+ T cell activity or infiltration, potentially leading to a less effective immune response against the tumor. This was consistent with findings that highlight the importance of CD8+ T cell responses in controlling tumor growth and improving patient outcomes in various cancers, including LC (43, 44). Similarly, memory CD4+ T cells and Th2 CD4+ T cells are essential for orchestrating the immune response. Memory CD4+ T cells help maintain long-term immunity, while Th2 cells are involved in humoral immunity and can influence the tumor microenvironment by modulating the activity of other immune cells. The negative correlation of PYGB with these CD4+ T cell subsets might indicated a suppression of helper T cell functions, which could impair the overall immune response to lung cancer (45, 46). Plasmacytoid dendritic cells (pDCs) and B cells, including plasma B cells, are also critical components of the immune system. pDCs are known for their role in producing type I interferons and activating other immune cells, while B cells contribute to antibody production and antigen presentation. The negative correlation of PYGB with these cell types suggested that PYGB might be involved in creating an immunosuppressive environment that hinders the activation and function of these cells, thereby facilitating tumor progression (47, 48).

Multiple immune infiltration analysis tools, such as CIBERSORT, EPIC, TIMER, MCP-Counter, and QuanTIseq were employed to validate these findings. Whether these immune cells play a crucial role in the poor prognosis of LC predicted by high PYGB expression warrants further investigation. Immune cell infiltration levels and tumor purity are inversely proportional, and immune cell infiltration levels are negatively correlated with Immune checkpoint inhibitors (ICI) response in multiple tumors (49, 50). Elevated expression of PYGB might diminish immune cell infiltration in LC, consequently influencing the prognosis of ICI therapy. These hypotheses undoubtedly necessitated additional experimental validation. Overall, the negative correlations between PYGB and these various immune cell types in LC highlighted the potential role of PYGB in modulating the immune landscape of the tumor microenvironment. Understanding these interactions could provide insights into new therapeutic strategies that target metabolic pathways to enhance anti-tumor immunity and improve patient outcomes.

We further investigated the relationship between PYGB expression and immune checkpoints, immune regulatory genes, as well as TMB and MSI, Notably, PYGB exhibited distinct immunoregulatory networks across LUAD and LUSC subtypes. In LUAD, PYGB expression showed robust positive correlations with immunosuppressive mediators (ADORA2A, CD276/B7-H3, TGFB1, IL12A) and protumorigenic cytokines (TNFSF9, IFNA1, CX3CL1, HMGB1, ENTPD1, TNFSF4), while displaying a marked negative association with the immune-inhibitory ligand VSIR (C10orf54). The expression of PYGB was significantly correlated with ADORA2A, a gene integral to the adenosine signaling pathway, which is critical for immunosuppression within the tumor microenvironment. This correlation implied that PYGB might contribute to the immunosuppressive milieu of LUAD through interactions with adenosine signaling pathways (51). Furthermore, PYGB expression was positively associated with CD276 (also known as B7-H3), an immune checkpoint molecule implicated in the inhibition of anti-tumor T-cell responses. The increased expression of CD276 in tumors is frequently linked to poor prognosis, suggesting that PYGB may play a role in modulating immune evasion strategies in LUAD (52). The association between PYGB and TGFB1, a pivotal cytokine in the regulation of immune responses and tumor progression, further emphasized the potential role of PYGB in fostering a tumor-supportive microenvironment. TGFB1 is recognized for its dual function in cancer, serving as a tumor suppressor during the initial stages and as a promoter of tumor progression in later stages (53). The positive correlation between PYGB and IL12A, a cytokine involved in T cell differentiation and the enhancement of cytotoxic activity, indicated that PYGB might influence immune cell activation and function in LUAD. This interaction could potentially impact the tumor’s capacity to evade immune surveillance (54). Furthermore, the relationship between PYGB and CD70, a member of the tumor necrosis factor (TNF) family, underscored its potential role in immune regulation. CD70 expression has been associated with immune escape mechanisms in various cancers, including LUAD, through its interaction with the CD27 receptor on T cells (55).

The observed positive correlation between PYGB and TNFSF9 (also known as 4-1BBL), a co-stimulatory molecule that enhances T-cell proliferation and survival, suggested that PYGB might be involved in modulating T-cell responses within the tumor microenvironment. This interaction could be pivotal for the development of effective immune responses against LUAD (56). Additionally, the association of PYGB with IFNA1, a type I interferon, implied its potential role in antiviral responses and immune modulation. Type I interferons are essential for the activation of immune cells and the initiation of anti-tumor immune responses (57). Moreover, the positive relationship between PYGB and CX3CL1, a chemokine involved in recruiting immune cells to the tumor site, indicated that PYGB might influence the infiltration of immune cells into the tumor microenvironment. CX3CL1 is recognized for its role in attracting cytotoxic T cells and natural killer cells, which are crucial for anti-tumor immunity (58). Furthermore, PYGB’s positive correlation with HMGB1, a protein involved in regulating inflammation and immune responses, suggested its potential involvement in the inflammatory processes associated with tumor progression. HMGB1 is recognized for its role in facilitating the recruitment of immune cells and the activation of pro-inflammatory pathways (59). The interaction between PYGB and ENTPD1 (also referred to as CD39), an ectoenzyme responsible for the hydrolysis of extracellular ATP and ADP, further underscored its involvement in modulating the immune microenvironment. ENTPD1 is implicated in the production of adenosine, which exerts immunosuppressive effects within the tumor microenvironment (51). The positive correlation between PYGB and TNFSF4 (also known as OX40L), a co-stimulatory molecule that enhances T-cell activation and survival, suggested its potential role in modulating T-cell responses and promoting anti-tumor immunity (60). In contrast, PYGB expression was inversely correlated with VSIR, an immunomodulatory receptor that inhibits T-cell effector function and maintains peripheral tolerance. This inverse correlation implied that PYGB might counteract the immunosuppressive effects mediated by VSIR, potentially enhancing immune activation in LUAD (53).

In LUSC, PYGB demonstrated strong positive linkages to T-cell exhaustion markers (HAVCR2/TIM-3, TIGIT), angiogenic factor VEGFB, and cytotoxic mediators (CD27, PRF1), but inversely correlated with PD-L1 (CD274) and the TNF superfamily member LIGHT (TNFSF14). The roles of HAVCR2/TIM-3 and TIGIT as immune checkpoints have been extensively investigated. These molecules are recognized for their immunosuppressive functions, often acting in conjunction with other immune checkpoints such as PD-L1. The expression of TIM-3 and its ligand, Galectin-9, has been identified in various malignancies, including cervical and vulvar cancers, indicating a potential role for TIM-3 checkpoint inhibition in combination with anti-PD-1/PD-L1 therapies (61). Co-expression of immune checkpoints, such as PD-L1 and TIM-3/TIGIT, has been correlated with poor overall survival in several cancer types, including esophageal squamous cell carcinoma, underscoring the potential of combination therapies targeting these checkpoints to enhance clinical outcomes (61). Similarly, TIGIT expression is associated with T-cell suppression and exhaustion, which predicts clinical outcomes and responses to anti-PD-1 therapies in follicular lymphoma (62). In LUSC, the inverse correlation between PYGB and both PD-L1 and TNFSF14 indicated a complex interaction between metabolic pathways and immune checkpoints. The expression of PD-L1, a critical immune checkpoint ligand, is influenced by various factors, including hypoxic conditions and inflammatory signals. For example, a high co-expression of PD-L1 and hypoxia-inducible factor-1α (HIF-1α) has been documented in pulmonary pleomorphic carcinoma, where it correlates with tumor necrosis and poor prognosis (63). Additionally, the role of angiogenic factors, such as vascular endothelial growth factor B (VEGFB), in modulating the tumor microenvironment and influencing immune checkpoint expression is an area of ongoing research. The interaction between angiogenesis and immune checkpoints presents a potential target for enhancing the efficacy of immunotherapies. Furthermore, the expression of CD27, a member of the tumor necrosis factor receptor superfamily, has been associated with immune modulation in cancer, with its ligand CD70 being regulated by hypoxia-inducible factors in renal cell carcinoma (64). The correlation between PYGB expression and immune checkpoints differed between LUSC and LUAD, suggesting the presence of distinct immune microenvironments, genomic characteristics, and immune evasion mechanisms specific to these LC subtypes (65). Understanding these variations is crucial for elucidating tumor biology and could inform the development of tailored immunotherapeutic approaches. The differential correlation patterns suggested that PYGB might play a pivotal role in orchestrating immune evasion mechanisms, thereby serving as a potential predictive biomarker or therapeutic target for modulating immune checkpoint pathways in LC immunotherapy.

Subsequent analysis of the correlation between PYGB expression and immune regulatory genes revealed significant associations with many immune regulatory genes in both LUAD and LUSC, with particularly strong correlations observed in LUAD. This suggested that PYGB might play a central role in shaping the tumor immune infiltration microenvironment in lung cancer, potentially highlighting its role as a therapeutic target for immunomodulatory strategies, and that further studies were needed to clarify the relationship.

Despite employing a range of experimental methodologies, including bioinformatics, cellular assays, and immunohistochemistry, it was imperatived to conduct a comprehensive analysis of the mechanisms underlying this phenomenon. Furthermore, additional experiments, such as animal trials, should be performed to provide robust evidence elucidating the *in vivo* role of PYGB. We had suggested future research paths, such as performing complementary *in vivo* and *in vitro* experiments to confirm the specific functions of PYGB in LC.

In summary, PYGB potentially contributed to the pathogenesis of LC by influencing both tumor cells and tumor-infiltrating immune cells. Increased expression of PYGB were associated with a poor prognosis in LC patients and facilitated the proliferation, invasion, and metastasis of LC cells. Additionally, PYGB might serve as a novel therapeutic target for the clinical management of LC and as a predictive marker for the efficacy of immunotherapy.

## Data Availability

The datasets presented in this study can be found in online repositories. The names of the repository/repositories and accession number(s) can be found in the article/**Supplementary Material**.

## Supplementary material

Supplementary Figure 1 PYGB protein expression analysis in LC. **(A)** PYGB protein expression in normal lung tissue (200× magnification); (**B)** PYGB protein expression in normal lung tissue (400× magnification); **(C)** PYGB protein expression in LUSC tumor tissue (200× magnification); (**D)** PYGB protein expression in LUSC tumor tissue (400× magnification); (**M):** Quantification of immunostains for PYGB by IOD analysis. ****p* < 0.001 PYGB, Brain glycogen phosphorylase; LC, lung cancer; LUSC, Lung squamous cell carcinoma.

Supplementary Table 1 Clinical characteristics of patients with LC. LC, lung cancer.
