# Supplementary material for: Aberrant expression of PYGB as a potential therapeutic target and its associations with immune cell infiltration in lung cancer
Source: Front Immunol. 2025 May 19;16:1536248. doi: 10.3389/fimmu.2025.1536248 (PMC12127346; doi:10.3389/fimmu.2025.1536248)
Supplement: Supplementary Table 1 — Clinical characteristics of patients with LC. LC, lung cancer. [file Table1.docx]

**Table S1**, Clinical characteristics of patients with lung cancer.

| Characteristic | No.of patients(%) |
| --- | --- |
| **n** | **15** |
| **Age, n (%)** |  |
| ≤55 | 3(20%) |
| >55 | 12 (80%) |
| **Gender, n (%)** |  |
| Male | 4 (26.7%) |
| Female | 11 (73.3%) |
| **Tumor size, n (%)** |  |
| ≦5cml | 6 (40%) |
| >5cm | 9(60%) |
| **Smoking, n (%)** |  |
| No | 9 (60%) |
| Yes | 6 (40%) |
| **Histological type, n (%)** |  |
| Squamous cell carcinoma | 12 (80%) |
| Adenocarcinoma | 3 (20%) |
| **Differentiation, n (%)** |  |
| well | 1 (6.6%) |
| Moderate | 10 (66.7%) |
| Poor | 4 (26.7%) |
| **Lymph node metastasis, n (%)** |  |
| No | 10 (56.7%) |
| Yes | 5 (33.3%) |
| **TNM stage, n (%)** |  |
| I-II | 8 (53.3%) |
| III-IV | 7 (46.6%) |
